# ‘FIFA 11 for Health’ for Europe. II: effect on health markers and physical fitness in Danish schoolchildren aged 10–12 years

**DOI:** 10.1136/bjsports-2016-096124

**Published:** 2016-04-29

**Authors:** Christina Ørntoft, Colin W Fuller, Malte Nejst Larsen, Jens Bangsbo, Jiri Dvorak, Peter Krustrup

**Affiliations:** 1 Department of Nutrition, Exercise and Sports, Copenhagen Centre for Team Sport and Health, University of Copenhagen, Copenhagen, Denmark; 2 Colin Fuller Consultancy Ltd, Sutton Bonington, UK; 3 FIFA Medical Assessment and Research Centre, Zurich, Switzerland; 4 Schulthess Clinic, Zurich, Switzerland; 5 Faculty of Life and Environmental Sciences, Sport and Health Sciences, University of Exeter, Exeter, UK

**Keywords:** Body composition, Cardiovascular, Children, Football, Non-communicable disease

## Abstract

**Objectives:**

To evaluate whether a modified ‘FIFA 11 for Health’ programme for non-communicable diseases had effects on body composition, blood pressure and physical fitness of Danish schoolchildren aged 10–12 years.

**Design:**

A cluster-randomised controlled study with 7 intervention and 2 control schools.

**Participants:**

546 Danish 5th grade municipal schoolchildren allocated to an intervention group (IG; n=402: 11.1±0.4 (±SD) years, 150.1±7.0 cm, 41.3±8.4 kg) and a control group (CG; n=144: 11.0±0.5 years, 151.2±7.8 cm, 41.3±9.0 kg).

**Intervention:**

As part of the physical education (PE) curriculum, IG carried out 2 weekly 45 min ‘FIFA 11 for Health’ sessions focusing on health issues, football skills and 3v3 games. CG continued regular school PE activities. Measurements of body composition, blood pressure at rest, Yo-Yo intermittent recovery level 1 children's test (YYIR1C), balance, jump and sprint performance were performed before and after the 11-week study period.

**Results:**

During the 11-week study period, systolic blood pressure (−3.5 vs 0.9 mm Hg), mean arterial blood pressure (−1.9 vs 0.4 mm Hg), body mass index (−0.02 vs 0.13 kg/m^2^) and body fat percentage (−0.83% vs −0.04%) decreased more (p<0.05) in IG than in CG. Within-group improvements (p<0.05) were observed in IG for 20 m sprint (4.09±0.29 to 4.06±0.28 s) and YYIR1C performance (852±464 to 896±517 m), but these changes were not significantly different from CG, and balance or jump performance remained unchanged in both groups.

**Conclusions:**

The modified ‘FIFA 11 for Health’ programme has beneficial effects on body composition and blood pressure for Danish schoolchildren aged 10–12 years, thereby providing evidence that this football-based health education programme can directly impact participants' cardiovascular health profile.

## Introduction

The ‘FIFA 11 for Health’ football-based 11-week health education programme consists of 90 min of football-related activities per week delivered by trained schoolteachers in an engaging and age-sensitive, gender-sensitive and culturally sensitive format. Ten health messages, each related to a communicable or non-communicable disease (NCD), are delivered on a weekly basis during the programme period, with each message linked to a football skill. The programme was first introduced in Africa in 2009 and has now been implemented in a total of 23 countries in Africa, Latin America, the Caribbean, South-east Asia and Oceania. The implementations are routinely monitored to assess the impact of the programme on children's health knowledge,^1^ and it has been shown that the programme is effective in increasing health knowledge in Africa,^2–4^ Mexico^5^ and Brazil.^6^ However, the effects of the programme on the children's health profile and physical fitness have so far not been evaluated.

When modifying the programme to the European context in order to focus on NCDs, modifications were made in relation to the physical activity content as well as the health messages.^7^ The modifications to the physical activity content were based on research on the fitness and health effects of football conducted at the University of Copenhagen. This research has shown that 6–16-week interventions with two weekly 30–60 min sessions of small-sided football provide marked broad-spectrum fitness and health improvements for participants across the lifespan.^8–11^ It has furthermore been shown that small-sided drills induce high heart rates (HRs), a high number of intense actions along with high involvement, technical success rates and training effects for boys and girls irrespective of body mass index (BMI), fitness level or prior experience with football.^12–15^ It was therefore decided to implement each 90 min session of the ‘FIFA 11 for Health’ programme over two separate 45 min periods conducted at least 1 day apart, and to include a high number of small-sided games and drills in order to ensure exercise periods with high intensity and high involvement for all participants. The modifications to the health messages, which were based on a rigorous examination of the health challenges in Europe arising from NCDs and an examination of successful and unsuccessful health campaigns in the Western world, resulted in a programme providing health education focusing on physical activity, nutrition and abuse as well as positive thinking and well-being. The effects of the programme on health knowledge and well-being are presented in an accompanying paper.^7^

The purpose of the present investigation was to evaluate whether the modified ‘FIFA 11 for Health’ programme for NCDs had effects on body composition, blood pressure and physical fitness in Danish schoolchildren aged 10–12 years.

## Methods

### Participants

**Figure 1:**
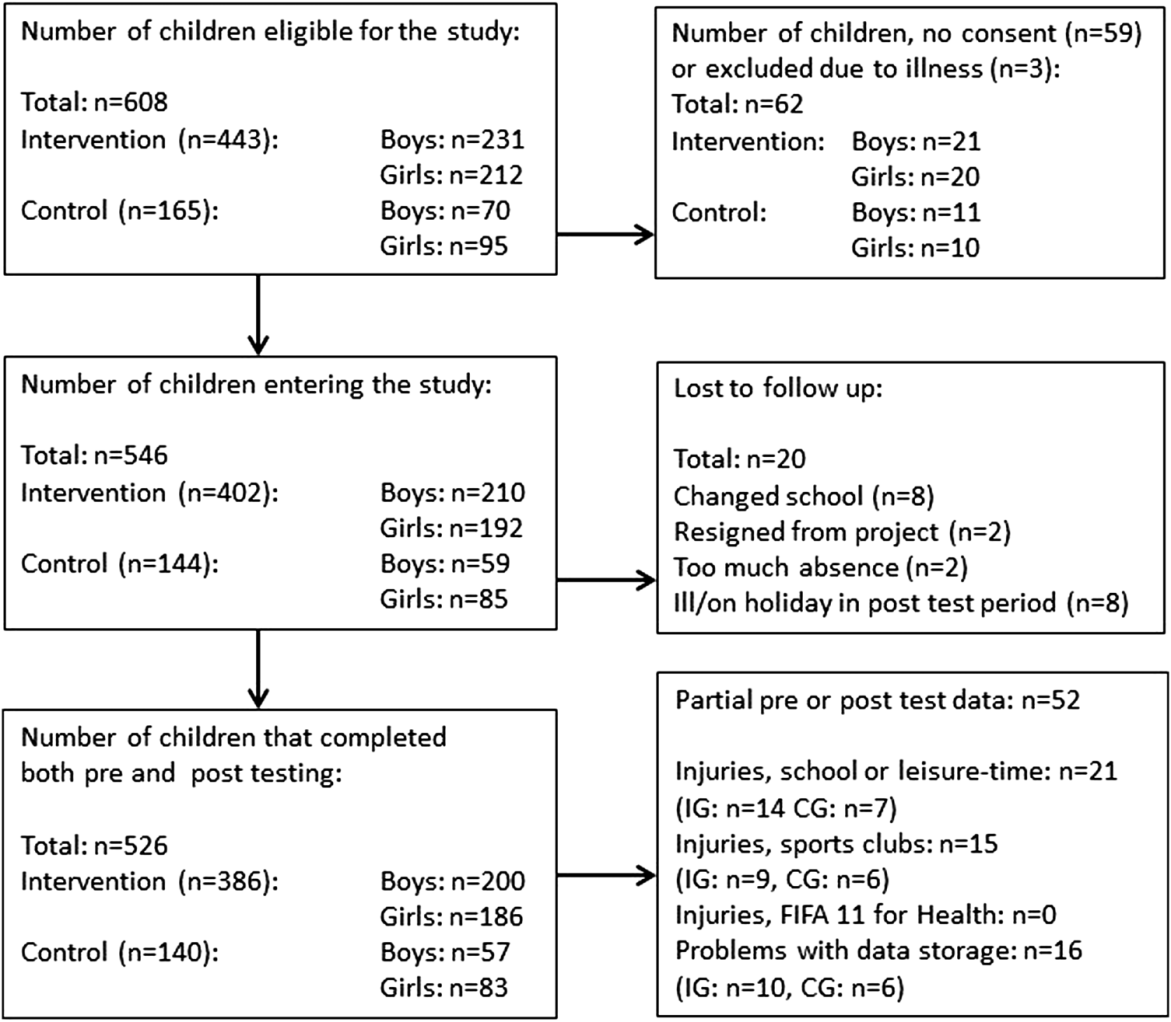
Study flow chart. CG, control group; IG, intervention group.

Eleven Danish schools that expressed an interest were contacted and provided with detailed information about the study. Of these, nine agreed to take part: five in the capital regions of the Frederiksberg and Copenhagen municipalities and four in the countryside regions of the Frederikssund and Roskilde municipalities. Each of the schools had 2–4 fifth grade classes for children aged 10–12 years; 546 pupils (boys: 269; girls: 277) in 26 classes agreed to participate in the study. The study was designed as a cluster-randomised controlled trial, with individual schools as clusters. Block randomisation of participating schools was performed with one school from the capital regions and one from the countryside regions assigned to the control group (CG) and four schools from the capital regions and three from the countryside regions assigned to the intervention group (IG). In total, 526 children completed both the preintervention and postintervention testing; of those, 386 were from the IG (age: 11.1±0.4 years, height: 150.1±7.0 cm, weight: 41.3±8.4 kg) and 140 from the CG (age: 11.0±0.5 years, height: 151.2±7.8 cm, weight: 41.3±9.0 kg); figure 1. The study was approved by the Committees on Biomedical Research Ethics for the Capital Region of Denmark (J.nr. H-15008117). Child assent and written informed parental consent were obtained for all participants.

During weeks 3–6 of the school year, tests were performed at the various schools. Participants completing each of the individual tests varied for a number of reasons (figure 1). Eight of the original participants did not complete the postintervention testing as they changed school (IG: n=7; CG: n=1), one from each group resigned from the study and two were excluded from IG due to too much absence from the intervention (figure 1). Eight children were ill or on holiday at either the preintervention or postintervention testing. Some of the children were unable to complete all tests preintervention or postintervention because of injuries sustained in their spare time or during playtime at school; some of the children sustained these injuries between test days 1 and 2. Other children had illnesses that prevented them from completing some of the tests (figure 1). Lastly, some data points were lost due to methodological problems (loss of transmission from the HR belt, data not stored correctly) (figure 1). No time-loss injuries were sustained during the ‘FIFA 11 for Health’ sessions. In total, 71% of the children (boys: 72%, girls: 70%) were sport club active, with 37% of the boys and 16% of the girls being football club members. The fraction of sport club active children was 68% in the IG and 75% in the CG.

### Design

**Figure 2:**
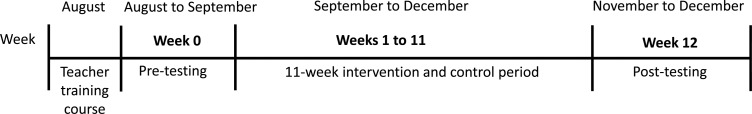
Timeline of the FIFA 11 for Health intervention in Denmark.

The study, which was carried out in the period August–December 2015, began with a 2½-day training course for teachers followed by a 13-week study period (weeks 0–12) at each school (figure 2). Week 0 was used for preintervention testing at both the intervention and control schools when the children completed the test battery. From weeks 1–11, the seven intervention schools completed 22 sessions during two scheduled 45 min physical education periods per week separated by at least 1 day. In this same time period, the two control schools undertook their normal physical education classes. Finally, in week 12, the children from both the intervention and control schools completed the same test battery as in week 0. In Denmark, it is obligatory for children to have an average of 45 min of physical activity in school per day. Each ‘FIFA 11 for Health’ session was undertaken during two of these designated 45 min periods. All tests were performed by trained university staff with the test leader being blinded to group allocation, with support by the schoolteachers.

### ‘FIFA 11 for Health’ programme

The overall structure, content and implementation procedure for the ‘11 for Health’ programme have been described previously in detail.^2^ ^3^ For the Danish project, the programme structure was retained but the content was modified: each session consisted of a 45 min play football period (teaching football skills and playing 3v3 football games) and a 45 min play fair period (teaching a health message and healthy behaviours related to an NCD) (table 1). The play football and play fair periods of each session were scheduled to take place within a single week but with at least 1 day between each period. Teachers from the participating schools, both intervention schools and control schools, were taken through a 2½-day interactive training course by FIFA Medical Assessment and Research Centre (F-MARC), staff members from the University of Copenhagen and football coaches from the Danish Football Association. This course provided participants with a detailed programme manual that itemised and described each aspect of the programme, outlined the programme's health-enhancing philosophy of physical activity linked with health education, demonstrated the football skills and discussed the health knowledge associated with each session of the programme. The training course also discussed the presentational skills required to deliver the ‘FIFA 11 for Health’ programme in an entertaining but informed way to children aged 10–12 years and finally tested participants' ability to deliver sessions using a series of teach-backs. During the delivery of the ‘FIFA 11 for Health’ programme within the schools, the headmaster was responsible for ensuring that the programme remained on schedule at each school and provided support to the teachers. One teacher usually delivered sessions for 20–25 children (mixed boys and girls).

**Table 1:** Overview of the modified ‘FIFA 11 for Health’ programme

Session	‘Play football’ activity	‘Play fair’ health topic	Issues addressed in session
1	Warming up	Play football	Prepare for exercise and sport
2	Passing	Respect others	Respect and help others and avoid bullying
3	Goalkeeping	Eat a balanced diet	Eat a full range of food types
4	Dribbling	Avoid drugs, alcohol and cigarettes	Avoid developing unhealthy addictions
5	Shielding	Be active	Walk, cycle, use the stairs in daily life
6	Controlling	Control your weight	Control the quantity of food eaten
7	Defending	Wash your hands	Develop good hygiene practices
8	Building fitness	Keep fit	Undertake sufficient vigorous exercise
9	Trapping	Drink water	Drink water and skimmed milk
10	Shooting	Think positively	Make friends and have a positive body image
11	Team working	Fair play	Review all health issues discussed in sessions 1–10

### Test battery

The test battery consisted of measurements of body composition (height, weight), resting blood pressure (mm Hg) and resting HR (bpm) along with a number of exercise and motor performance tests. The tests were completed over two consecutive days with a requirement that no physical activity be performed on the day before test day 1. Test day 1 included two 20 m sprints, two maximal horizontal jumps and the Yo-Yo intermittent recovery level 1 children's test (YYIR1C). Before the tests were started, the children had a standardised warm-up consisting of two sets of exercises 1, 4, 12.1 and 13 of the FIFA 11+ warm-up programme.^16^ Test day 2 consisted of measuring resting blood pressure and HR, height and weight and postural balance.

### Body composition

Height was measured with a 0.1 cm precision using a Tanita Leicester Transportable altimeter (Tanita, Amsterdam, the Netherlands). Weight, per cent body fat and lean body mass were measured using an InBody 230 multifrequency body composition analyser (Biospace, California, USA) as described by Karelis *et al*.^17^ Specifically, the participants were weighed barefoot in light clothes, between 9:00 and 10:30, after having breakfast before 8:00. Data output, as calculated by the manufacturer’'s algorithm, included fat mass (kg), body fat (%) and lean body mass (kg). The InBody 720 has been validated with dual-energy X-ray absorptiometry (DXA) on primary schoolchildren with interclass correlation coefficients (ICC) for fat percentage and lean mass of 0.94–0.99,^18^ ^19^ and the InBody 230 has been validated on adults with ICCs for fat performance and lean body mass of 0.98–0.99.^17^

### Resting blood pressure and HR

Arterial blood pressure was measured with the participants in the supine position following at least 10 min of supine rest in a quiet room. Blood pressure was recorded as the average of three measurements on the left upper arm by an automatic blood pressure monitor (M6 HEM-7223-E, Omron, Illinois, USA); the cuff size was adjusted to the arm as appropriate. Resting HR was measured simultaneously by the automatic blood pressure monitor and in 15 s intervals over the entire resting period using a HR belt (POLAR Team2 system, Polar Electro Oy, Kempele, Finland). The lowest HR value was used as the test result.

### Twenty-metre sprint test

After the warm-up, the children performed two 20 m maximal sprints with at least 2 min of recovery between sprints. All sprints started from a standing position and were timed using two ports of light sensors (Witty Microgate, Bolzano, Italy) placed at 0 m (positioned 30 cm in front of the standing start position) and at 20 m: the best time recorded was noted as the test result.

### Horizontal jump length

The warm-up included instructions about completing the squat jump from the akimbo position. The children were placed with their toes just behind a line with their feet parallel and shoulder width apart and standing erect; after flexing the knees to the squat position and holding the position for at least 2 s, the children jumped as far as they could: the distance from the start line to the heel position was measured. Each child had two attempts; if they did not perform two correct jumps, they were allowed an additional attempt: the longest jump was noted as the test result.

### Yo-Yo intermittent recovery level 1 children's test

The test was performed indoors on one half of a wooden-floor handball court in accordance with previous studies.^20^ ^21^ The test consisted, in short, of two 16 m shuttle runs back and forth between cones placed 16 m apart (at the start/finish line and turning line) at progressively increasing speeds, interspersed by 10 s of jogging after each running bout, around a cone placed 4 m behind the start/finish line. The total duration of the test varied from 2 to 12 min. The HR was recorded at 1 s intervals using a Polar team system 2 (Polar Electro Oy, Kempele, Finland) during the tests to determine HR_peak_, that is, the highest value registered during each test. The children's HR_peak_ had to be within 10 bpm to ensure that they were running to exhaustion both times. The test–retest coefficient of variation of the YYIR1C has been shown to be 13% for children aged 9–16 years.^22^

### Flamingo balance test

Postural balance was assessed using a single-leg Flamingo balance test.^23^ The children were instructed to stand on one foot on a 3 cm wide, 5 cm high and 50 cm long metal bar with their eyes open, holding the contralateral leg at the ankle joint for 1 min. The children were permitted to move their arms and non-standing leg to assist balancing. The number of falls was counted and used as a measure of postural balance. After choosing their preferred leg, the children were given two attempts before the number of falls was counted.

### Statistics

Data are reported as means (SD), unless otherwise stated. Possible differences in baseline values between IG and CG were tested using analyses of variance (ANOVA). Within-group changes for IG and CG were tested using Student's paired t test. Between-group differences in δ values were tested using ANOVA. Significance was accepted at p<0.05.

## Results

### Sample population

At baseline, there were no significant differences in the age, height, weight, BMI, fat percentage, lean mass, resting HR, postural balance and jump performance of participants in IG and CG, whereas blood pressure and YYIR1C performance was higher (p<0.05) and sprint performance was lower (p<0.05) in IG compared with CG (table 2).

**Table 2:** Body composition before (pre) and after (post) the 11-week study period for the CG and the IG participating in two weekly 45 min FIFA 11 for Health football sessions

	Control		Intervention		Δ		Difference
	Pre (n=124)	Post (n=124)	Pre (n=343)	Post (n=343)	CG (n=124)	IG (n=343)	IG vs CG
Height (cm)	151.4±7.8	152.6±7.9†	150.1±7.0	151.7±7.0†	1.2±1.8	1.6±1.3	0.4‡
Weight (kg)	41.7±8.4	42.7±8.7†	41.2±8.2	42.1±8.4†	1.0±1.5	0.9±1.3	−0.1
BMI (kg/m^2^)	18.1±2.5	18.2±2.6	18.2±2.7	18.2±2.7	0.13±0.75	−0.02±0.59	−0.15‡
Body fat (%)	21.4±6.9	21.2±7.3	21.5±7.5	20.7±7.7†	−0.04±1.83	−0.83±2.30	−0.80‡
Lean mass (kg)	17.0±3.1	17.6±3.2†	16.8±2.8	17.5±3.0†	0.5±0.9	0.6±0.7	0.1

Data are presented as means±SD.

*Significantly different from control at the same measuring moment.

†Significant within-group difference.

‡Significant between-group difference.

BMI, body mass index; CG, control group; IG, intervention group.

### Body composition

During the 11-week study period, body fat percentage decreased more (p<0.05) in IG than in CG (−0.83±2.30% vs −0.04±1.83%) and the change score in BMI was lower (p<0.05) in IG compared with CG (−0.02±0.59 vs 0.13±0.75 kg/m^2^) (table 2). Body height increased more (p<0.05) in IG than in CG (1.6±1.3 vs 1.2±1.8 cm) (table 2). Lean body mass (0.6±0.7 and 0.5±0.9 kg) and total body mass (0.9±1.3 and 1.0±1.5 kg) increased (p<0.05) in IG and CG but with no differences between the groups (table 2).

### Resting blood pressure and HR

During the 11-week study period, the systolic blood pressure decreased more (p<0.05) in IG (−3.5±11.2 mm Hg) than in CG (0.9±10.2 mm Hg), whereas the change in diastolic blood pressure was not significantly different in IG compared with CG (−1.2±7.2 vs 0.1±7.9 mm Hg, p=0.09) (table 3). Thus, mean arterial blood pressure decreased more (p<0.05) in IG compared with CG (−1.9±7.5 vs 0.4±7.9 mm Hg). No significant changes occurred in resting HR in either IG (−0.7±10.0 bpm) or CG (0.3±8.0 bpm; p>0.05) (table 3).

**Table 3:** Blood pressure and resting HR before (pre) and after (post) the 11-week study period for the CG and the IG participating in two weekly 45 min FIFA 11 for Health football sessions

	Control		Intervention		Δ		Difference
	Pre (n=140)	Post (n=140)	Pre (n=354)	Post (n=354)	CG (n=140)	IG (n=354)	IG vs CG
SBP (mm Hg)	103±11	104±8	109±10*	106±9†	0.9±10.2	−3.5±11.2	−4.3‡
DBP (mm Hg)	61±6	61±6	64±6*	62±6†	0.1±7.9	−1.2±7.2	−1.3 (p=0.09)
MAP (mm Hg)	75±7	76±6	79±7*	77±6	0.4±7.9	−1.9±7.5	−2.3‡
Resting HR (bpm)	70±10	70±11	72±10	71±10	0.3±8.0	−0.7±10.0	−1.0

Data are presented as means±SD.

*Significantly different from control at the same measuring moment.

†Significant within-group difference.

‡Significant between-group difference.

CG, control group; DBP, diastolic blood pressure; HR, heart rate; IG, intervention group; MAP, mean arterial pressure; SBP, systolic blood pressure.

### Physical fitness

During the 11-week period, within-group improvements (p<0.05) were observed in IG in YYIR1C performance (5.2%, 852±464 to 896±517 m) and 20 m sprint performance (4.09±0.29 to 4.06±0.28 s), whereas no change occurred in CG (1±236 m and 0.00±0.16 s, respectively) (table 4). However, the change scores in YYIR1C and 20 m sprint performance were not significantly different between IG and CG (table 4).

**Table 4:** Physical performance before (pre) and after (post) the 11-week study period for the CG and the IG participating in two weekly 45 min FIFA 11 for Health football sessions

	Control		Intervention		Δ		Difference
	Pre (n=123–140)	Post (n=123–140)	Pre (n=329–369)	Post (n=329–369)	CG (n=123–140)	IG (n=329–369)	IG vs CG
Yo-Yo IR1C (m)	731±383	731±427	852±464*	896±517*†	1±236	44±296	44
20 m sprint (s)	3.98±0.28	3.98±0.32	4.09±0.29*	4.06±0.28*†	0.00±0.16	−0.03±0.15	−0.03
Flamingo balance (falls, n)	19.3±9.0	20.4±10.3	18.0±9.6	18.3±9.4*	1.1±8.4	0.3±8.8	−0.9
Horizontal jump length (cm)	120±20	119±20	118±17	117±18	−1±11	−1±13	−0.6

Data are presented as means±SD.

*Significantly different from control at the same measuring moment.

†Significant within-group difference.

CG, control group; IG, intervention group; IR1C, intermittent recovery level 1 children's test.

During the 11-week period, no changes in performance were observed in the Flamingo balance test or the horizontal jump length in either group (table 4).

## Discussion

The main findings of the present study were that the modified ‘FIFA 11 for Health’ programme focusing on NCDs had positive effects in terms of fat percentage, BMI and blood pressure for Danish schoolchildren aged 10–12 years. Together with the intervention effects on health knowledge and well-being presented in an accompanying paper, the pilot study in Denmark has revealed that this 11-week intervention football-based health education programme can be used effectively within a school's curriculum to induce improvements in psychosocial and physiological health profile along with increases in health knowledge.

It is evident that precursors of adult cardiovascular disease (CVD) begin in childhood and that paediatric obesity has an important influence on overall CVD risk, tracks to later life and is associated with impaired life quality and early mortality.^24–27^ Recent studies have defined and evaluated the importance of six CVD risk factors, including systolic blood pressure, fat percentage, aerobic fitness, homeostasis model assessment (HOMA) score, cholesterol and triglycerides.^28–30^ These risk factors have been shown to cluster and to be associated with the level of physical activity in children from 9 years of age and older.^28^ ^30^ ^31^ In the present investigation, three of the six CVD risk factors were evaluated (ie, blood pressure, fat percentage and aerobic fitness) and it was shown that the ‘FIFA 11 for Health’ programme had positive effects on blood pressure and fat percentage when compared with the CG that continued with its regular physical education regime. Moreover, within-group improvements were also observed in the YYIR1C, which has been shown to be correlated with maximal oxygen uptake and thereby aerobic fitness.^20^

During the 11-week intervention period, systolic blood pressure and mean arterial pressure, respectively, were reduced by as much as 4.4 and 2.3 mm Hg, respectively, in the ‘FIFA 11 for Health’ IG compared with the CG. This effect is greater than the average effects on systolic and mean arterial blood pressure of 1.3 mm Hg reported in meta-analyses of physical activity intervention studies on children aged 6–12 years.^32^ For adults, the risk of cardiovascular mortality and morbidity is reduced by 13% for every 5 mm Hg reduction in systolic blood pressure,^33^ and although no equivalent risk calculation has been made in children, it is well established that a reduction in blood pressure of this magnitude, if sustained, is associated with less arterial stiffness and a decreased atherosclerosis progression rate in adulthood.^34^ ^35^ Interestingly, the blood pressure effects were present for both the non-sport club active children as well as the sport club active children, but the effects were larger for the 29% of children who were not attending sport club activities in their leisure time (5.5 and 2.8 mm Hg). Although the childhood fitness and health profile is generally very high in Denmark, a polarisation has been identified over the past decade, with 98% of the children football club members and only 72% of non-sport club active children achieving the national recommendations for physical activity.^36^ ^37^ On that basis, it is of interest that the non-sport club active children gained a large benefit from the school-based ‘FIFA 11 for Health’ programme.

Although about half of the school-based physical activity interventions lasting 10–52 weeks have been shown to have positive effects on aerobic fitness and other fitness components, few of them have had positive effects on body composition and fat percentage.^32^ ^38^ ^39^ In this study, BMI was reduced by 0.15 kg/m^2^ and body fat percentage by 0.8% within the IG during the 11-week period of the ‘FIFA 11 for Health’ compared with the CG, with similar effects for the sport club active and non-sport club active children. It is noteworthy that this reduction was achieved with 90 min of activity per week over an 11-week intervention period, which is much less than in previous investigations observing similar reductions in BMI and fat percentage in interventions with higher training volume and longer duration, for example, 330 min of activity over 9 months.^40^ In three previous football intervention studies with normal weight and overweight children aged 8–12 years playing small-sided football for 3×40 and 3×60 min/week for 10 and 12 weeks, respectively, marked positive effects were observed in heart function and physical fitness, whereas no changes were observed in BMI or fat percentage.^13^ ^41^ ^42^ Since changes in fat percentage and blood pressure can be obtained by diet manipulations, physical activity interventions and other lifestyle changes and as additive effects can be achieved with combined interventions,^43^ it may be speculated that there are additional effects of the ‘FIFA 11 for Health’ programme over and above the football activity itself. Thus, the on-pitch education on health knowledge related to food and fluid intake and importance of high-intensity physical training as well as everyday life physical activities such as walking and cycling could have influenced the children's behaviour during the 11-week intervention period. However, further studies are needed to elucidate whether the increases in health knowledge observed following the ‘FIFA 11 for Health’ programme^7^ have resulted in the desired behavioural changes towards better dietary habits and/or more physical activity.

In this study, within-group improvements were observed for the ‘FIFA 11 for Health’ IG in 20 m sprint performance (1%) and YYIR1C performance (5%) with no differences in the effects for the sports club active and non-sport club active children. The improvement in sprint performance is similar to previous short-term football studies, whereas the improvement in YYIR1C performance is lower than in previous investigations showing 10–25% performance increments in 6–12 weeks with two or three 30–60 min football sessions per week,^12^ ^13^ ^41^ a difference that is probably not related to the higher baseline value in the IG in this study, as the previous study with 2×30 min football per week over 6 weeks showed similar increases for children with low and high YYIR1C baseline values.^12^ Since the ‘FIFA 11 for Health’ programme combines health education with physical activity, the average exercise intensity and activity level may not have been as high as in the previous small-sided football studies, and the improvements in Yo-Yo performance for young schoolchildren have been shown to be closely related to the time spent in the highest aerobic intensity zone.^13^ Nonetheless, the modified ‘FIFA 11 for Health’ programme focusing on NCDs did include two weekly 45 min sessions rather than one 90 min session and did include at least 30 min/week of small-sided games (2v2 to 5v5) with at least 20 min in the play football session and at least 10 min in the play fair session (see table 1). This study suggests that this protocol was sufficient to induce multiple effects on cardiorespiratory health and also to provide some improvements in sprint performance and intermittent exercise performance. However, the latter findings should be interpreted with caution as the change scores were not significantly different from the CG that continued its regular physical education curriculum. It would therefore be relevant to determine the intervention effects on cardiovascular, metabolic and musculoskeletal fitness effects for subgroups with low fitness, obesity, high cholesterol or other CVD risk factors in the upcoming large-scale nationwide study in Denmark starting August 2016, and also to evaluate the intensity of each of the 22 sessions in the modified ‘FIFA 11 for Health’ programme with HR measurements and locomotor analyses.

In conclusion, the modified ‘FIFA 11 for Health’ programme focusing on NCDs has beneficial effects on body composition and blood pressure for Danish schoolchildren aged 10–12 years, whether or not they are sport club active. Considering that risk factors for CVDs, such as high systolic blood pressure and fat percentage along with low aerobic fitness, are clustering from the age of 9, this study provides evidence that the modified programme provides a short-duration, football-based health education programme that can have a direct impact on children's cardiovascular health profile.

**What are the findings?**

- The ‘FIFA 11 for Health’ programme has been modified to focus on non-communicable diseases and has now been evaluated in the European context.
- The ‘FIFA 11 for Health’ in Europe was shown to provide beneficial effects on blood pressure and body composition for Danish schoolchildren aged 10–12 years.
- The modified programme with two weekly 45 min sessions with football-related drills, high-intensity small-sided games and elements from the 11+ warm-up programme had a high attendance and a low injury rate.

**How might it impact on clinical practice in the future?**

- Evidence-based school-based physical activity concepts with positive effects on risk factors for cardiovascular diseases are warranted in the European context.
- The ‘FIFA 11 for Health’ in Europe can easily be implemented in the European school system as it incorporates two weekly 45 min sessions and is being taught by schoolteachers.
- The use of drills and small-sided games that can be conducted in small areas on a variety of indoor and outdoor surfaces provides potential for safe and easy implementation.
- The ‘FIFA 11 for Health’ programme will be delivered in Denmark from August 2016 along with pilot phase projects in Greenland and the Faroe Islands and there are plans for expansion of the programme from 2017 to 2019.

## Supplementary Material

Web abstract
