# Supplementary material for: ‘FIFA 11 for Health’ for Europe. II: effect on health markers and physical fitness in Danish schoolchildren aged 10–12 years
Source: Br J Sports Med. 2016 Apr 29;50(22):1394–9. doi: 10.1136/bjsports-2016-096124 (PMC5136709; doi:10.1136/bjsports-2016-096124)
Supplement: Web abstract [file bjsports-2016-096124-s1.pdf]

Abstrakt

Formål At evaluere om det justerede "FIFA 11 for Health" program mod livsstilssygdomme har en effekt på kropssammensætning, blodtryk og fysisk kapacitet for 10-12-årige danske skolebørn.

Design Et cluster-randomiseret kontrolleret studie med 7 interventionsskoler og 2 kontrolskoler.

Deltagere 546 danske folkeskoleelever i 5. klasse blev efter lodtrækning fordelt i en "FIFA 11 for Health" interventionsgruppe (IG; n=402: 11.1±0.4 år, 150.1±9.7 cm, 41.3±8.4 kg) og en kontrolgruppe (CG; n=144: 11.0±0.5 år, 151.2±7.8 cm, 41.3±9.0 kg).

Intervention Interventionsgruppen gennemførte to ugentlige 45-min sessioner med FIFA 11 for Health" i 11 uger, med fokus på sundhedstemaer, boldøvelser og småspil 2v2, 3v3 og 4v4. Undervisningen blev gennemført på udendørs eller indendørs fodboldbaner af lærere som havde været på kursus gennemført i samarbejde mellem FIFA's forskningsenhed F-MARC, Dansk Boldspil-Union og Center for Holdspil og Sundhed på Københavns Universitet. Kontrolgruppen fortsatte deres almindelige idræts- og bevægelsesundervisning. Før og efter interventionsperioden blev gennemført målinger af kropssammensætning, blodtryk og præstation i Yo-Yo IR1C, balance-, hoppe- og sprinttests.

Resultater Efter 11 ugers intervention var der en positiv effekt ($p<0.05$) på det systoliske blodtryk (-3.5 vs 0.9 mmHg), gennemsnitligt arterietryk (-1.9 vs 0.4 mmHg), BMI (-0.02 vs 0.15 kg/m^2) og fedtprocent (-0.83 vs -0.04%) i "FIFA 11 for Health"-interventionsgruppen i forhold til kontrolgruppen. Der blev tillige observeret forbedringer ($p<0.05$) i interventionsgruppen i præstation ved 20-m sprint (4.09±0.29 til 4.06±0.28 s) og Yo-Yo IR1C testen (852±464 til 896±517 m), men disse ændringer var ikke signifikant forskellige fra kontrolgruppen. Balance- og hoppepræstation var uforandret i begge gruppe i interventionsperioden.

Konklusioner Den justerede udgave af "FIFA 11 for Health" programmet viste sig at have positive effekter på kropssammensætning og blodtryk hos 10-12-årige danske skolebørn. Disse fund dokumenterer at den vestlige version af "FIFA 11 for Health" - som supplement til de kortlagte effekter på sundhedsviden og social trivsel - har en direkte effekt på deltagernes kardiovaskulære sundhedstilstand.
